# Three Yemeni Siblings With Johanson-Blizzard Syndrome: A Case Report and Literature Review

**DOI:** 10.7759/cureus.103579

**Published:** 2026-02-14

**Authors:** Shatha J Abukammas, Abobakr Abdelgalil, Mohamed Abdelmaksoud Shazly, Marwah M Alghanmi, Taqiyah Z Sheriff

**Affiliations:** 1 Medicine and Surgery, Batterjee Medical College, Jeddah, SAU; 2 Pediatrics, Kasr Al-Ainy Faculty of Medicine, Cairo University, Cairo, EGY; 3 Pediatrics, King Abdulaziz University Hospital, Jeddah, SAU; 4 Medicine, Batterjee Medical College, Jeddah, SAU

**Keywords:** consanguinity marriage, craniofacial abnormalities, exocrine pancreatic insufficiency, johanson-blizzard syndrome, ubr1 gene

## Abstract

Johanson-Blizzard syndrome (JBS), also known as UBR1-related disorder, is a very rare autosomal recessive disorder caused by pathogenic variants in the UBR1 gene and characterized by significant phenotypic variability. The condition is known to be mainly characterized by craniofacial abnormalities, exocrine pancreatic insufficiency, growth retardation, and sensorineural hearing loss. We describe three affected siblings from a consanguineous Yemeni family with JBS. Two brothers suffered from profound symptoms resulting in infant death, which included failure to thrive, exocrine pancreatic dysfunction, anemia, hypoalbuminemia, aplasia cutis congenita, and cardiomyopathy, which was only present in one sibling. The third sibling, who is still alive, is a one-year-old girl who presented with vomiting, diarrhea, failure to thrive, and marked facial dysmorphic features, including hypoplastic alae nasi, a beaked nose, brachycephaly, and a fifth-finger anomaly, without significant visceral malformations. Genome analysis of the affected sibling revealed a homozygous missense mutation in the UBR1 gene, following the American College of Medical Genetics and Genomics (ACMG) guidelines. Moreover, the familial form, consanguinity, and typical presentation are highly suggestive of a diagnosis of JBS. The current case report draws attention to the significant variability of JBS within families and, once again, emphasizes the need for precise clinical assessment in order to make a diagnosis, especially when molecular testing might be equivocal in resource-poor environments. Early multidisciplinary supportive care and genetic counseling are pivotal for optimizing patient survival and minimizing the rate of recurrence within affected kindreds. A narrative review of the literature was conducted to contextualize the clinical findings and highlight intrafamilial phenotypic variability.

## Introduction

Johanson-Blizzard syndrome (JBS) is a rare hereditary disorder caused by mutations in the UBR1 gene that disrupt the N-end rule pathway. Fewer than 100 cases of JBS have been reported since its discovery. The condition affects both genders equally and is often associated with positive consanguinity. In Saudi Arabia, five cases have been documented. The first involved a child born to consanguineous parents in 2008. In 2024, four additional cases were identified: three siblings born to first-degree cousins and a fourth, unrelated individual. Other reported cases from the Middle East include two from Bahrain (one consanguineous as of 2024), one from Yemen (consanguineous in 2011), and one reported case from Syria (consanguineous in July 2025). JBS is characterized by craniofacial dysmorphisms, exocrine pancreatic insufficiency, hypoplasia or aplasia of the alae nasi, sensorineural hearing loss, mental and growth retardation, hypothyroidism, genitourinary anomalies, imperforate anus, and dental problems. Phenotypic expression varies considerably, even among siblings. In addition to reporting this familial case, a narrative review of the literature was conducted to contextualize the observed phenotypic variability. Relevant articles were identified through searches of PubMed and Google Scholar using the terms "Johanson-Blizzard syndrome," "UBR1 mutation," and "exocrine pancreatic insufficiency." The review was non-systematic and aimed to highlight clinical features, intrafamilial variability, and diagnostic challenges reported in prior cases. The objective of this report is to highlight the marked intrafamilial variability of Johanson-Blizzard syndrome and to emphasize the importance of clinical diagnosis in the setting of a UBR1 variant of uncertain significance. Early recognition of Johanson-Blizzard syndrome is critical, as delayed diagnosis may result in severe malnutrition, recurrent infections, and fatal complications due to untreated exocrine pancreatic insufficiency and multisystem involvement [1-4].

## Case presentation

We reported a one-year-old Yemeni female who presented with green-colored diarrhea and recurrent vomiting. She experienced recurrent vomiting of all ingested food. Her clinical history revealed that she had no surgeries or allergies, and her vaccinations were up to date for her age. She was born via spontaneous vaginal delivery (SVD) with a birth weight of 2.6 kg, had no neonatal intensive care unit (NICU) admission, and was discharged in good condition. Both of her parents were middle-aged, and there was positive consanguinity, as they are first-degree cousins. She has a three-year-old healthy sister. Her two brothers, diagnosed with Johanson-Blizzard syndrome, died at the ages of six months and nine months. The clinical characteristics of the three affected siblings are summarized in Table 1.

**Table 1:** Clinical characteristics of the three siblings with Johanson-Blizzard syndrome. VUS: variant of uncertain significance

Characteristics	Sibling 1 (male)	Sibling 2 (male)	Sibling 3 (female)
Age at presentation	Infancy	Infancy	1 year
Outcome	Deceased (6 months)	Deceased (9 months)	Alive
Consanguinity	Yes	Yes	Yes
Failure to thrive	Yes	Yes	Yes
Diarrhea/vomiting	Yes	Yes	Yes
Exocrine pancreatic insufficiency	Yes	Yes	Yes
Hypoalbuminemia	Yes	Yes	Yes
Anemia	Yes	Yes	Yes
Edema	Yes	Yes	Yes
Craniofacial anomalies	Yes	Yes	Yes
Hypoplastic nasal alae	Yes	Yes	Yes
Aplasia cutis congenita	Yes	Yes	No
Cardiomyopathy	Yes	No	No
Developmental delay	Yes	Yes	Yes
Genetic testing (UBR1)	Homozygous missense variant (VUS)	Homozygous missense variant (VUS)	Homozygous missense variant (VUS)

She had frequent hospital admissions due to repeated infections and recurrent edema, receiving several antimicrobials, frequent albumin infusions, and blood transfusions. The observed anemia was considered multifactorial and most likely related to chronic malabsorption and nutritional deficiency. Although Diamond-Blackfan anemia and other bone marrow failure syndromes were considered, they were deemed less likely based on the clinical course and available laboratory findings. Bone marrow examination was not performed. She is taking multivitamins and minerals and receiving multidisciplinary follow-up from ophthalmology, gastroenterology, genetics, and general medicine.

On examination, she had dysmorphic features (brachycephaly, a prominent occiput, hypoplastic nasal alae, a small beak-like nose, and fused fifth toes bilaterally). She was vitally stable. Neurologically, she was alert and active with equal and reactive pupils and intact power, tone, and reflexes. At the time of evaluation, the proband demonstrated developmental delay, predominantly affecting gross motor and social milestones. Formal standardized developmental assessment tools were not applied; however, the delay was clinically evident on examination. In the cardiovascular system (CVS), she had normal S1 and S2 and no murmurs, a capillary refill of 3 s, and intact peripheral pulses. The respiratory examination showed equal breath sounds bilaterally with no added sounds. Her abdomen was distended, soft, lax, with no organomegaly. The throat was clear, and the tympanic membranes were intact bilaterally. The genital area was normal for a female. Laboratory investigations are summarized in Table 2. System-based evaluation included thyroid function tests, liver enzymes, renal function tests, and abdominal ultrasound. Thyroid and renal parameters were within normal limits, liver enzymes were unremarkable, and abdominal ultrasound did not reveal structural abnormalities. Cardiomyopathy in sibling 1 was diagnosed during infancy based on echocardiographic findings. Screening echocardiography was subsequently performed on sibling 2 and the proband and revealed no structural or functional cardiac abnormalities. Formal audiological assessment using otoacoustic emissions or auditory brainstem response testing was not performed at the time of evaluation due to resource limitations. This is acknowledged as a limitation of the present report.

**Table 2:** Laboratory results of a patient with Johanson-Blizzard syndrome. Hb: hemoglobin; WBC: white blood cell; Na: sodium; K: potassium; Cl: chloride; BUN: blood urea nitrogen

Tests	Results	Reference range
Hb (g/dL)	10.9	10.2-16
WBC count (×10³/µL)	9.73	4.5-17.5
Neutrophils (×10³/µL)	4.47	1-8.5
Lymphocytes (%)	51.9	20-40
Platelets (×10³/µL)	494	150-450
Na (mmol/L)	142	135-145
K (mmol/L)	3.2	3.5-5.0
Cl (mmol/L)	114	98-106
Creatinine (µmol/L)	22	53-115
BUN (mmol/L)	2.8	2.5-7.8
Magnesium (mmol/L)	0.9	0.7-1.0
Phosphate (mmol/L)	0.91	0.8-1.5
Albumin (g/L)	16	35-50
Iron (µmol/L)	8.9	10-30
Ferritin (ng/mL)	286.7	30-400
Stool analysis	Salmonella spp.	Not applicable

Abdominal ultrasonography and echocardiography were performed as part of the clinical evaluation. No brain imaging studies or skeletal surveys were performed during the course of management, as these investigations were not clinically indicated at the time and were limited by the patient’s overall condition and resource availability.

Genetic testing using whole-exome sequencing was performed for all three affected siblings and identified the same homozygous missense variant in the UBR1 gene in each case. Genetic analysis using whole-exome sequencing identified a homozygous missense variant in the UBR1 gene (NM_174916.2:c.4526T>G; p.Leu1509Arg), located in exon 41 of 47. The variant is absent from major population databases and affects a highly conserved amino acid residue. In-silico prediction tools (PolyPhen-2, SIFT, and MutationTaster) suggest a deleterious effect. According to the American College of Medical Genetics and Genomics (ACMG) guidelines, this variant is classified as a variant of uncertain significance (Class 3). Parental segregation analysis was not performed. Although the same homozygous missense variant was identified in all three affected siblings, molecular confirmation remains limited by the classification of this variant as a variant of uncertain significance (VUS). Therefore, the diagnosis in this family is supported by a combination of molecular findings and characteristic clinical features.

According to the American College of Medical Genetics and Genomics (ACMG) guidelines, the identified UBR1 variant was classified as a variant of uncertain significance (VUS). This classification was based on limited available evidence, including rarity in population databases (PM2) and supportive in silico predictions suggesting a deleterious effect on protein function (PP3). However, the absence of functional studies, segregation analysis, and previously reported pathogenic associations precluded definitive pathogenic classification.

## Discussion

Johanson-Blizzard syndrome (JBS) is a rare multisystem disorder with considerable phenotypic variability, which presents challenges for early recognition and diagnosis. Even within families, affected individuals may exhibit varying severity of clinical manifestations, underscoring the complex interplay among the underlying UBR1 mutations, modifier genes, and environmental influences. This variability underscores the importance of detailed clinical assessment and vigilance in populations with high rates of consanguinity [2,3,5].

Previously reported pathogenic variants in the UBR1 gene associated with Johanson-Blizzard syndrome are predominantly truncating or splice-site variants, which are typically linked to severe phenotypes. In contrast, the variant identified in the present case is a missense change, located in exon 41, and has not been previously reported in association with the disease. This difference may partly explain the observed phenotypic variability and underscores the importance of correlating molecular findings with clinical features.

Genetic analysis identified a homozygous missense variant in the UBR1 gene, classified as a Variant of Uncertain Significance (VUS, Class 3) according to the American College of Medical Genetics and Genomics guidelines. From a strict molecular standpoint, this finding alone cannot confirm pathogenicity. Therefore, the diagnosis in this family was primarily clinical, supported by the characteristic phenotype, intrafamilial recurrence, and consanguinity, with molecular findings serving as supportive rather than definitive evidence.

Marked intrafamilial variability was observed among the three affected siblings, who shared core clinical features yet differed in severity and clinical course. This variability highlights the heterogeneous expression of Johanson-Blizzard syndrome even within the same family and emphasizes the need for individualized clinical assessment.

The broad-spectrum manifestations in JBS, encompassing craniofacial dysmorphisms, ectodermal abnormalities, exocrine pancreatic insufficiency, and variable neurodevelopmental involvement, reflect the multifaceted role of UBR1 in cellular proteostasis and tissue-specific development. Experimental models, such as the *Caenorhabditis elegans* ubr-1 system, suggest that UBR1 deficiency disrupts metabolic and neurotransmission pathways, providing mechanistic insights into observed neurological phenotypes and highlighting avenues for future research [6,7].

Despite the genetic basis being increasingly well-characterized, clinical heterogeneity remains a defining feature of JBS, with variable penetrance and expressivity observed even among individuals carrying similar mutations. This reinforces the necessity for a comprehensive, multidisciplinary approach to diagnosis and management, particularly in settings where molecular testing may not be immediately available. Early recognition based solely on phenotypic assessment can guide timely interventions and inform genetic counseling, thereby improving patient outcomes [1,2,3,5,7].

Gastrointestinal involvement is among the most common and clinically significant features of JBS. Exocrine pancreatic insufficiency leads to malabsorption, steatorrhea, and failure to thrive, typically apparent in the first year of life. Almashraki et al. described a child with diarrhea and malnutrition who improved with pancreatic enzyme supplementation, whereas Isa et al. reported growth improvement in a Bahraini child after early initiation of pancreatic enzyme therapy. Our patient exhibited vomiting, diarrhea, steatorrhea, and failure to thrive, suggesting malabsorption. Her clinical stabilization with supportive care aligns with previous observations [1,2].

Hematologic features in JBS are less well-characterized but include anemia and, rarely, Diamond-Blackfan anemia. Our patient’s mildly transfusion-responsive anemia, along with prior transfusions at four and 10 months, may reflect either nutritional deficiency or intrinsic hematologic pathology. The etiology of anemia in this patient remains unclear. While nutritional deficiency related to malabsorption is the most likely explanation, bone marrow failure syndromes, including Diamond-Blackfan anemia, were considered. However, the absence of macrocytosis, lack of persistent transfusion dependence, and clinical stability argue against an underlying marrow failure disorder [8].

Neurological and auditory involvement significantly affects development and quality of life. Sensorineural deafness is common and often requires early cochlear implantation. Alwadee et al. showed marked improvement in communication following cochlear implantation in JBS patients. In our patient, neurological assessment revealed intact power, tone, and reflexes, consistent with reports of preserved gross motor milestones despite metabolic vulnerability. Mild developmental delay may emerge over time, reflecting variable penetrance [1,2,3,6,9].

Skin and ectodermal abnormalities are frequently observed, ranging from scalp defects to nail hypoplasia and sparse hair. Our patient exhibited a nonspecific rash treated with zinc and bilaterally fused fifth toes, illustrating subtle yet diagnostically relevant cutaneous and skeletal disorders. Nutritional status was compromised, evidenced by edema and hypoalbuminemia, underscoring the need for systematic monitoring and supplementation [1,2,3,5]. Infectious complications, such as Salmonella-positive diarrhea, highlight vulnerability due to malabsorption and nutritional compromise. Prompt antibiotic therapy, hydration, and electrolyte correction facilitated recovery [1,2].

Family history and consanguinity are particularly pertinent in JBS because of its autosomal recessive inheritance pattern. Our patient was born to first-degree cousins, and both prior siblings were diagnosed with JBS and deceased in infancy. The first brother had an anomaly of the anus, anemia, aplasia cutis congenita, diarrhea, edema, exocrine pancreatic insufficiency, failure to thrive, global developmental delay, hypoalbuminemia, and underdeveloped nasal alae. The second brother had anemia, aplasia cutis congenita, diarrhea, edema, exocrine pancreatic insufficiency, failure to thrive, global developmental delay, hypoalbuminemia, and underdeveloped nasal alae. These observations reinforce the clinical diagnosis and highlight the importance of genetic counseling for future pregnancies. The recurrence in siblings emphasizes variable expressivity and penetrance [1,2,3,5,10].

This case underscores the continued importance of careful phenotypic assessment in rare genetic syndromes, particularly in consanguineous populations and in settings where molecular confirmation may be inconclusive. Classical craniofacial features, exocrine pancreatic insufficiency, growth and developmental delay, and recurrence among siblings remain central to establishing the diagnosis and guiding clinical management.

Differential diagnoses include Shwachman-Diamond syndrome, Pearson marrow pancreas syndrome, and ectodermal dysplasia syndromes, which share features of pancreatic insufficiency or dysmorphic facial features but lack classical JBS traits. The combination of hypoplastic nasal alae, beaked nose, fifth-toe fusion, episodic anemia, and family history strongly points toward JBS [1,2,3,5,8,11].

Management is primarily supportive and requires a multidisciplinary approach. Core elements include pancreatic enzyme replacement therapy, nutritional support, audiologic evaluation, and regular hematologic monitoring. In our case, care consisted of intravenous fluids, electrolyte correction, nutritional and vitamin support, and consultations with ophthalmology, gastroenterology, genetic, and metabolic specialists, consistent with current evidence-based recommendations [1,2,8,9,12].

Prognosis in JBS is highly variable and depends on pancreatic insufficiency, associated anomalies, recurrent infections, and access to multidisciplinary care. Early recognition and management of nutrition, infections, and hearing loss are critical. In our case, stabilization had led to short-term remission. Ongoing monitoring of growth, development, and complications is essential. Given autosomal recessive inheritance and affected siblings, genetic counseling is recommended [1,2,3].

Several differential diagnoses were considered, including Shwachman-Diamond syndrome and other syndromic causes of exocrine pancreatic insufficiency. However, the presence of characteristic craniofacial features, congenital anomalies, severe growth failure, and the clinical constellation observed in this patient and affected siblings favored a diagnosis of Johanson-Blizzard syndrome. Additionally, the absence of definitive hematologic or skeletal findings consistent with alternative diagnoses further supported this clinical impression. Limitations of this report include the classification of the identified UBR1 variant as a Variant of Uncertain Significance (VUS), incomplete standardized developmental and audiological assessments, and the absence of functional validation studies.

## Conclusions

Johanson-Blizzard syndrome remains a clinically and genetically heterogeneous disorder, and cases with inconclusive molecular findings, such as variants of uncertain significance (VUS), continue to highlight the importance of detailed phenotypic recognition, particularly in regions with a high degree of consanguinity. This case emphasizes that typical craniofacial features, ectodermal anomalies, and pancreatic involvement may be sufficiently diagnostic when complemented by an appropriate family history and sibling recurrences. Early diagnosis is crucial for initiating supportive management and providing appropriate genetic counseling, especially when previous siblings were affected or deceased. Reporting such cases enhances understanding of the phenotypic spectrum of JBS and underscores the need for access to genetic testing and long-term multidisciplinary follow-up. Long-term data on growth, developmental milestones, and hearing outcomes are unavailable. Nevertheless, this case provides valuable clinical insight in a resource-limited setting, where JBS may be underdiagnosed. The classical phenotypic presentation, familial occurrence, and consanguinity provide strong evidence for the diagnosis. Reporting cases from underrepresented regions contributes to global knowledge and enhances early recognition in consanguineous populations.
